# Feasibility and Performance of Loop-Mediated Isothermal Amplification Assay in the Diagnosis of Pulmonary Tuberculosis in Decentralized Settings in Eastern China

**DOI:** 10.1155/2019/6845756

**Published:** 2019-01-22

**Authors:** Zhongdong Wang, Haiyan Sun, Zhisheng Ren, Bai Xue, Jie Lu, Huaqiang Zhang

**Affiliations:** ^1^Qingdao Municipal Center for Disease Control and Prevention, Qingdao, China; ^2^Beijing Key Laboratory for Pediatric Diseases of Otolaryngology, Head and Neck Surgery, MOE Key Laboratory of Major Diseases in Children, Beijing Pediatric Research Institute, Beijing Children's Hospital, Capital Medical University, National Center for Children's Health, Beijing, China

## Abstract

Early diagnosis is essential for the control and prevention of tuberculosis (TB). The objective of this study was to investigate the feasibility and performance of loop-mediated isothermal amplification (LAMP) in the diagnosis of pulmonary TB in county-level microscopy centers in Qingdao, Eastern China. A total of 523 presumptive TB patients were consecutively recruited between July 2017 and April 2018, and 22 patients were excluded from the analysis. Of 102 culture-positive cases, TB-LAMP identified 91 cases, demonstrating a sensitivity of 89.2%. In comparison, the sensitivity of routine smear microscopy was 69.6% (71/102), which was significantly lower than that of TB-LAMP (*P*=0.001). In addition, TB-LAMP sensitivities in smear-positive and smear-negative samples were 98.6% and 67.7%, respectively. In conclusion, our data demonstrate that TB-LAMP outperforms conventional smear microscopy in TB diagnosis, which could be used as an alternative method for smear microscopy in resource-limited settings in China.

## 1. Introduction

Tuberculosis (TB), caused by* Mycobacterium tuberculosis* complex (MTBC), remains the major public health concern worldwide [1]. In 2017, an estimated 10 million people developed active TB diseases and 1.6 million people died from this disease [1]. Early diagnosis and immediate initiation of treatment are essential component of the WHO's END Tuberculosis Strategy [2]. Sputum microscopy is the primary method for diagnosing pulmonary tuberculosis in low-income and middle-income countries [3]. It is rapid, of low cost, and specific in areas where there is a high burden of TB, whereas a major shortcoming of conventional microscopy is its low relatively sensitivity, especially for individuals co-infected with HIV [4]. Mycobacterial culture always requires long incubation time despite yielding satisfactory sensitivity [4]. There is an urgent need for accurate and timely diagnosis that limits morbidity, reduces costs, and improves patients' outcome.

Nucleic acid amplification technique (NAAT) provides speed and sensitivity for the detection of pathogen [5]. A number of NAAT assays have been invented to address the need for rapid and sensitive diagnosis of TB in clinical practice [2]. However, the expensive initial investment in the equipment and high cost of cartridge limit the use of NAAT in routine diagnosis of TB patients [6]. Recently, loop-mediated isothermal amplification (LAMP) has been developed by Eiken Chemical Company Ltd. (Tokyo, Japan) for detection of MTBC from clinical specimens [7]. In addition, the results of TB-LAMP can be interpreted through visualization with the naked eyes, which makes it more suitable for use in developing countries where TB is epidemic. Considering the promising performance reported by several researchers, WHO endorsed TB-LAMP for use as a replacement to smear microscopy or as a follow-on test to smear microscopy in smear-negative specimens for diagnosis of pulmonary TB in adults [1]. Despite having an international policy recommendation, the clinical studies conducted in different settings showed obvious diversity in the sensitivity of TB-LAMP to detect MTB from sputum specimens [4, 5, 8], indicating that it is necessary to evaluate the performance of TB-LAMP prior to the widespread roll-out of this assay. The objective of this study was to investigate the feasibility and performance of LAMP in the diagnosis of pulmonary TB in county-level microscopy centers in Qingdao, Eastern China.

## 2. Materials and Methods

### 2.1. Ethical Approval

The study protocol was approved by the Institute Ethics Committee of Qingdao Center for Disease Control and Prevention. All patients participating in this study provided written informed consent.

### 2.2. Study Design

A prospective evaluation study was carried out at five county-level microscopy centers in Qingdao, Eastern China. Qingdao is the largest coastal city of Shandong Province, with population prevalence of tuberculosis of 202/100, 000. Between July 2017 and April 2018, consecutive individuals with suspected pulmonary TB were recruited in this study. Patients were eligible if they had cough for 2 or more weeks and presence of any clinical symptoms, i.e., fever, chest pain, night sweats, and weight loss. Demographic and clinical characteristics were collected at the time of enrolment. Sputum was collected from each patient and then submitted to laboratory for examinations.

### 2.3. Laboratory Procedures

Smear microscopy was performed and reported according to National Tuberculosis Control Programme guidelines of China [9]. Sputum sample was smeared directly on a slide and subjected to Ziehl-Neelsen staining for acid fast bacilli and examined by experienced laboratory staff. Then, 60 *μ*L of sputum was used for the TB-LAMP assay and the remainder of the specimen was decontaminated with each volume of 4% NaOH for 15 minutes and inoculated onto modified Löwenstein-Jensen (L-J) medium according to previous report [8]. The inoculated L-J slants were incubated at 37°C and monitored weekly for bacterial growth for 8 weeks. The positive cultures on the slants were further identified by biochemical tests. Briefly, paranitrobenzoic acid (PNB) was incorporated into the L-J medium at a final concentration of 500 *μ*g/mL. The growth on L-J medium containing PNB indicates that the bacilli belong to nontuberculous mycobacteria.

The TB-LAMP assay was performed according to the manufacturer's instructions. Briefly, 60 *μ*L of sputum sample was pipetted into heating tubes and incubated at 90°C for 5 min. The purified DNA was eluted from the absorbent tube and transferred into injection caps. After mixing with lyophilized reagents, the amplification mixture was incubated at 67°C for 40 min. The final results were interpreted using ultraviolet fluorescence detection. The turnaround time of TB-LAMP was 60 min, and no more than 14 specimens could be handled per batch.

### 2.4. Statistical Analysis

Using the conventional culture as the gold standard for TB diagnosis, the sensitivity, specificity, positive predictive value (PPV), and negative predictive value (NPV) of the TB-LAMP assay were calculated, respectively. Pearson chi-square test was used to compare the sensitivity between the tests. In addition, the kappa statistic was conducted to gauge the strength of agreement between TB-LAMP and mycobacterial culture. Values of the kappa coefficient higher than 0.75 indicated excellent agreement. All statistical analysis was performed with SPSS software version 20.0 (SPSS Inc., Chicago, IL).

## 3. Results

**Figure 1:**
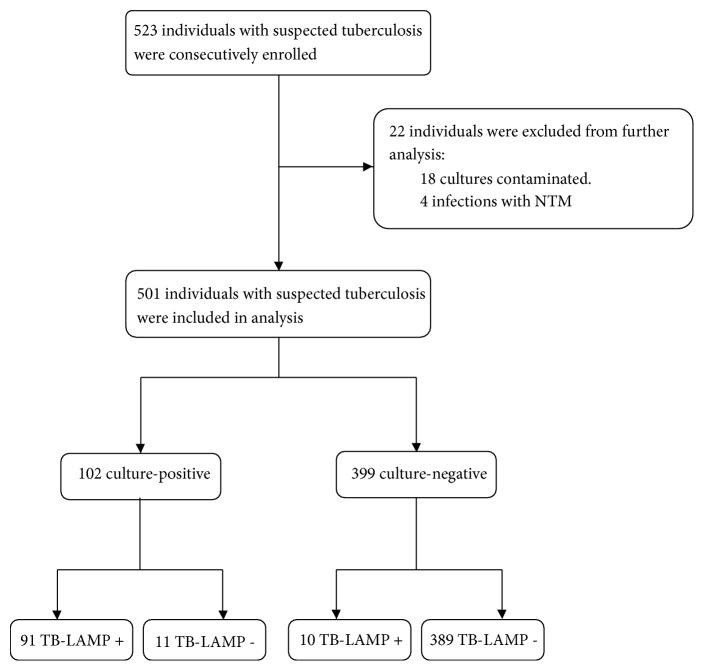
Patient enrollment.

A total of 523 pulmonary presumptive TB patients were consecutively recruited in the present study. Of these, 22 patients were excluded from the analysis, including 16 of culture contamination and 6 of nontuberculous mycobacteria infection. Of the 501 samples analyzed for the study, 332 (66.3%) were from males and 169 (33.7%) from females, with age range from 18 to 76 years (Figure 1).

Culture was positive for the* M. tuberculosis* complex in 102 cases. Of these culture-positive cases, TB-LAMP identified 91 cases, demonstrating a sensitivity of 89.2% (91/102, 95% CI: 83.2%~95.2%). In comparison, 71 of 102 culture-positive samples were detected by the routine smear microscopy, demonstrating a sensitivity of 69.6% (92/102, 95% CI: 60.7%~78.5%), which was significantly lower than that of TB-LAMP (*P*=0.001). In addition, TB-LAMP identified 389 of 399 culture-negative cases, resulting in a specificity of 97.5% (389/399, 95% CI: 96.0%~99.0%). The statistical analysis indicated that the results of TB-LAMP and mycobacterial culture showed high consistency, with a Kappa value of 0.870 (Table 1).

We also analyzed the performance of TB-LAMP according to the smear microscopy results. As shown in Table 1, TB-LAMP sensitivity in smear-positive and culture-positive samples was 98.6% (70/71, 95% CI: 95.9%~100.0%). In addition, twenty-one of 31 smear-negative, culture-positive samples were detected by TB-LAMP, yielding a sensitivity of 67.7% (21/31, 95% CI: 51.3%~84.2%). Statistical analysis revealed that TB-LAMP showed significantly better sensitivity in smear-positive, culture-positive samples than in smear-negative, culture-positive samples (*P*<0.001). Out of 10 “false-positive” cases determined by TB-LAMP, 2 (20.0%, 2/10) patients were smear-positive.

## 4. Discussion

The Eiken TB-LAMP assay demonstrated significantly higher sensitivity over smear microscopy in detecting TB cases at county-level microscopy centers in China. Our findings are in agreement with earlier reports on TB-LAMP evaluations which showed that TB-LAMP could identify nearly all smear-positive TB cases and approximately 67% of smear-negative TB cases [6, 8]. We also found that TB-LAMP sensitivities for smear-negative TB cases varied across previous studies (25% to 85%) [5, 6, 10]. One important explanation for this may be due to the different mycobacterial methods used as gold standard among various evaluations. There is now strong evidence demonstrating that MGIT system improves the yield to detection of MTB compared to conventional L-J media [11, 12]. Hence, the application of less sensitive mycobacterial culture method may be associated with the relative higher sensitivity of TB-LAMP in detecting smear-negative, culture-positive patients. Another reason for this disparity may be related to the paucibacillary nature of patients with HIV and TB coinfection [13]. The low number of MTB bacilli in sputum among HIV patients may lead to the “false negative” results by TB-LAMP. In agreement to our hypothesis, a recent study by Nakiyingi and colleagues reported that TB-LAMP could only identify one-quarter of smear-negative culture positive patients in a high HIV prevalence setting [5]. Therefore, the detection limit of TB-LAMP should be further improved to address the need for the molecular diagnostics among TB/HIV coinfected patients.

In addition to the sensitivity, TB-LAMP outperforms smear microscopy in several aspects. First, TB-LAMP could distinguish fairly well between nontuberculous mycobacteria (NTM) and MTB, thereby avoiding inappropriate anti-TB treatment for patients infected with NTMs. A systematic review demonstrated that the prevalence of NTM infections among tuberculosis suspects was 6.3% in China [14]. Similarly, nearly 5% of culture-positive patient affected NTM in our study; hence, the application of TB-LAMP will bring additional benefit for these individuals with presumptive TB. Second, infrastructure remains a concern at microscopy centers [6]. Ideally, only a heater block is required for TB-LAMP assay. On the basis of our experience, the technicians with no molecular experience could perform this assay after one-week training. Therefore, these advantages meet the criteria in term of equipment and human resource for microscopy centers. Third, no contamination events were recorded using the TB-LAMP assay in this study, suggesting the promising feasibility of TB-LAMP in the resource-limited settings.

In conclusion, our data demonstrate that TB-LAMP outperforms conventional smear microscopy in the diagnosis of MTB from individuals with presumptive TB. In addition, the practical requirement of TB-LAMP in infrastructure and training makes it more accessible in the laboratories with resource-limited settings. Further studies on cost-effective analysis of TB-LAMP are urgently needed to apply TB-LAMP as an alternative method for the diagnosis of TB.

## Figures and Tables

**Table 1:** Performance of TB-LAMP for the diagnosis of pulmonary tuberculosis.

Method	Diagnostic performance measure %(95% CI)^a^					
	Sensitivity			Specificity	PPV	NPV
	S+C+	S-C+	C+			
TB-LAMP	98.6	67.7	89.2	97.5	90.1	97.3
	(95.9~100.0)	(51.3~84.2)	(83.2~95.2)	(96.0~99.0)	(84.3~95.9)	(95.6~98.9)

^a^PPV: positive predictive value; NPV: negative predictive value; S: smear; C: culture; CI: confidence interval.

^b^Kappa value=0.870 (TB-LAMP vs. Culture).

## Data Availability

The data used to support the findings of this study are available from the corresponding author upon request.
